# Garlic (*A. sativum* L.) alliinase gene family polymorphism reflects bolting types and cysteine sulphoxides content

**DOI:** 10.1186/s12863-015-0214-z

**Published:** 2015-05-22

**Authors:** Jaroslava Ovesná, Katarína Mitrová, Ladislav Kučera

**Affiliations:** Crop Research Institute, Drnovská 507/73, 161 06 Prague-Ruzyně, Czech Republic

**Keywords:** *Allium sativum* L., Garlic, Alliinase, Polymorphism, Molecular marker, Bolting type

## Abstract

**Background:**

Alliinase is an important enzyme occurring in *Allium* species that converts precursors of sulfuric compounds, cysteine sulfoxides into a biologically active substance termed allicin. Allicin facilitates garlic defense against pests and produces health-promoting compounds. Alliinase is encoded by members of a multigene family that has not yet been sufficiently characterized, namely with regard to the copy numbers occurring within the genome and the polymorphisms among the family members.

**Results:**

We cloned 45 full-length alliinase amplicons of cultivar (cv.) Jovan. Sequence analyses revealed nine different sequence variants (SVs), confirming the multilocus nature of this gene family. Several mutations in exons, mainly occurring in the first exon coding for vacuolar signal peptide, were found. These results enabled us to identify sequences with putatively modified vacuole-targeting abilities. We found additional sequence variants using partial amplicons. We estimated that the minimum number of gene copies in the diploid genome of the investigated cultivar was fourteen. We obtained similar results for another three cultivars, which differed in bolting type and place of origin. The further identification of high degree of polymorphisms in the intron regions allowed us to develop a specific polymerase chain reaction assay capable to capture intron length polymorphism (ILP). This assay was used to screen 131 additional accessions. Polymorphic data were used for cluster analysis, which separated the bolting and non-bolting garlic types and those with high cysteine-sulfoxide contents in a similar way as AFLP analysis in previous study. These newly developed markers can be further applied for the selection of desirable garlic genotypes.

**Conclusions:**

Detailed analysis of sequences confirmed multigenic nature of garlic alliinase. Intron and exon polymorphism analysis generated similar results as whole genome variability assessed previously by AFLP. Detected polymorphism is thus also associated with cysteine-sulphoxide content in individual genotypes. ILP markers capable to detect intron polymorphisms were newly developed. Developed markers could be applied in garlic breeding. Higher genetic variability found in bolting genotypes may indicates longer period of their sexual propagation in comparison with nonbolting genotypes.

**Electronic supplementary material:**

The online version of this article (doi:10.1186/s12863-015-0214-z) contains supplementary material, which is available to authorized users.

## Background

The value of garlic (*Allium sativum* L*.*) as a crop has been recognized since ancient times. It is estimated to have been cultivated for over 5.000 years. Overall of these years, garlic has been used as a food, condiment and medicine by many cultures in Asia and the Mediterranean region [1]. Garlic has been considered to be valuable due to its antibacterial, antioxidant, anticancer and cholesterol-lowering effects [2].

Botanically, garlic belongs to the genus *Allium* in the family *Alliaceae*, which includes important vegetable crops, such as onion (*Allium cepa* L*.*), leek (*Allium ampeloprasum* L*.*) and shallot (*Allium ascalonicum* L*.*). Garlic (*Allium sativum* L.) is a clonally propagated diploid plant (2n = 16) [1].

*Allium* species typically contain a high concentration of non-protein sulfur amino acids that are responsible for their health-promoting features. One of the classes of these non-volatile sulfur secondary metabolites, S-alk(en)yl-L-cysteine sulfoxides, which are also known as diallylthiosulfinates, are responsible for the characteristic aroma of these crops. The compound alliin is the most common in garlic, while isoalliin is prevalent in onion. In an intact cell, sulfoxides are stored in the cytoplasm, and the hydrolytic enzyme alliinase is located in the vacuoles [3]. If a cell is damaged by pests or crushing, the vacuolar enzyme alliinase is released (alliin:lyase EC.4.4.1.4) which induces the conversion of alliin into allicin. This enzyme belongs to a family of lyases, and more specifically, a class of carbon-sulfurlyases. Within several seconds, this enzyme transforms alliin into allicin via the exceptionally reactive intermediate, sulfenic acid (R-SOH). Pyruvate and ammonium ion are by-products of this reaction. Afterwards, two molecules of sulfenic acid condense, forming allicin. Allicin, which is absent in intact bulbs, is the main component of freshly prepared garlic homogenate [4]. Many health benefits associated with garlic can be attributed to thiosulfinates, especially allicin [5].

This enzyme is a homodimeric glycoprotein formed from two identical subunits of 51.1 kDa each that contains four glycosylation sites [6, 7]. There are ten cysteine residues per alliinase monomer, eight of which form four disulfide bridges and two of which are free thiols. The residues Cys368 and Cys376 form an S − S bridge near the C-terminus, which plays an important role in maintaining both the rigidity of the catalytic domain and the substrate − cofactor relative orientation [8]. The activity of this enzyme depends on the reaction conditions, such as the pH, temperature and ion concentrations [9]. This enzyme can be found in other *Allium* sulfur-containing species (e.g. onion, leek, shallot, or chive).

The first *Allium* alliinase protein and cDNA sequences were published in 1992 by Van Damme et al. [10]. Since then, sequences originating from several species have been described and published, including several specific to leaf, bulb and root tissues [11–13]. Its coding sequence has been reported to be approximately 2.200 nucleotides long, coding for a 486-aminoacid polypeptide. The conformity of the deduced amino acid sequences of the leaf and bulb or root alliinases has been estimated not to exceed 72 % [12]. To date (August 2013), a total of 101.344 *Allium sativum* L. nucleotide sequences have been identified, of which 21.636 ESTs (Expressed Sequence Tags) have been collected from an in-house cDNA library, and 287 have been obtained from a genomic survey sequence (GSS) database of linear DNAs. Only four complete mRNA sequences and 104 partial alliinase sequences have been found. Alliinase is encoded by members of a multigene family, with variable number of members, which has not been sufficiently investigated.

Our work aimed to characterize polymorphisms in exon and intron sequences within alliinase gene family and its effects upon putative functionality of respective proteins. Further we investigated whether the data allow for the estimation of gene family members number. As we recently have characterized a set of garlic clones with respect to cysteine sulfoxides content and genetic diversity, as assessed by AFLP [14] showing that cysteine sulfoxides content is associated with genetic background of individual garlic clones. We investigated in addition whether the same applies for polymorphisms within alliinase gene family and cysteine sulfoxides content.

## Results

### Alliinase family DNA sequence analysis

The entire genomic sequences encompassing the introns and exons of alliinase family members from the 5′ UTR to the 3′ UTR captured from cv. Jovan (Czech bolting garlic) were amplified by primers covering the entire sequence (ALL_total_ Table 1) and then cloned. The resulting 45 clones were sequenced and analyzed to assess the polymorphisms within this gene family. Altogether, nine different DNA sequences were obtained and called sequence variants (SVs). The SVs differed in total nucleotide number and in nucleotide order due to insertions, deletions, transversions, and transitions. SV1, representing the [GenBank: Z12622] sequence obtained from the NCBI database, was used as a reference. Out of the newly identified SVs, six putative ORFs were of the same size, spanning 1.461 nucleotides from the start to the stop codon. SV8 was one nucleotide shorter and SV9 was three nucleotides longer than the others. Similar polymorphisms were further revealed in 68 DNA sequences resulting from the amplification of partial overlapping alliinase sequences (ALL_parts_) from cvs. Japo, Jovan, Djambu1 and landrace Marhfeld. These genotypes represented different garlic types (hardneck, softneck, and semibolters) and places of origin. These results thus confirmed that the sequence coding for alliinase (S-alk(en)yl-L-cysteine sulfoxidelyase, EC 4.4.1.4) consisted regulary of 5 exons and 4 introns regardless garlic bolting type.

**Table 1:** List of primer pairs, used for amplification of alliinase gene from Allium sativum L.

primer	sequence	annealing temperature
FWD-ALL_total_	5´ F TAATTAGCTATGGTGGAGTCT	63 °C
REV-ALL_total_	3´ R ATCAGACCGAGACGGCCT	63 °C
FWD-ALL_part1_	5´ 1F TAATTAGCTATGGTGGAGTCTTACA	65 °C
REV-ALL_part1_	3´ 1R GACCCCTACTCCAAACACAAT	65 °C
FWD-ALL_part2_	5´ 2F AGCACAAGGAAGCCAGTGCAG	65 °C
REV-ALL_part2_	3´ 2R TGGTGCCTTTCTGCGTTTTCA	65 °C
FWD-ALL_part3_	5´ 3F GATACGTGTGGGCCGGAAATG	65 °C
REV-ALL_part3_	3´ 3R AATGAAAGGAACGACGGGAGGC	65 °C

All analyzed sequences contained a start codon at a position corresponding with the 13^th^ nucleotide of the previously published sequence [GenBank: Z12622] (SV1). This first AUG translation start codon was in an optimal Kozak context (GCC(A/G)CC**AUG**G), with guanine at −3 and +4. The translation that began from this start codon yielded a putative 486-aa (amino acid) peptide with a 28-aa vacuolar signal peptide predicted for the secretory pathway characteristic for alliinase. We identified several alternative downstream in-frame translation start codons. Translation initiation from alternative translation start codons is expected to be presumably less frequent, and in addition, the resulting putative peptides would posses altered (shortened or otherwise) vacuolar signal peptides.

Moreover, a 1-nt deletion was detected in exon I of SV9 just before the alternative translation start codon, giving rise either to a frame shift and changes in the amino acid sequence of the propeptide or the use of a second AUG. If the first AUG is used as a regular start codon, translation results in the premature insertion of a stop codon, resulting in a truncated 16-aa peptide. The alternative ORF from the second downstream translation start codon yielded an N-truncated SV9 preprotein that was 473aa long. The remaining N-terminal 15-aa sequence did not fulfill the criteria of a signal peptide as assessed by Target 1P1 and Signal IP, suggesting that the putative SV9 protein sequence did not represent the vacuolar-targeted enzyme. Other identified downstream AUGs were not in optimal contexts (adenine at −3 and cytosine at +4 or adenine at −3 and thymine +4).

The insertion of three nucleotides was identified in SV6, SV7, SV8, SV9 (if the alternative ORF was considered) and SV10, leading to the insertion of the amino acid serine after Asn^33^in the propeptide. No effects on the signal peptide were predicted according to *in silico* analysis.

**Fig. 1:**
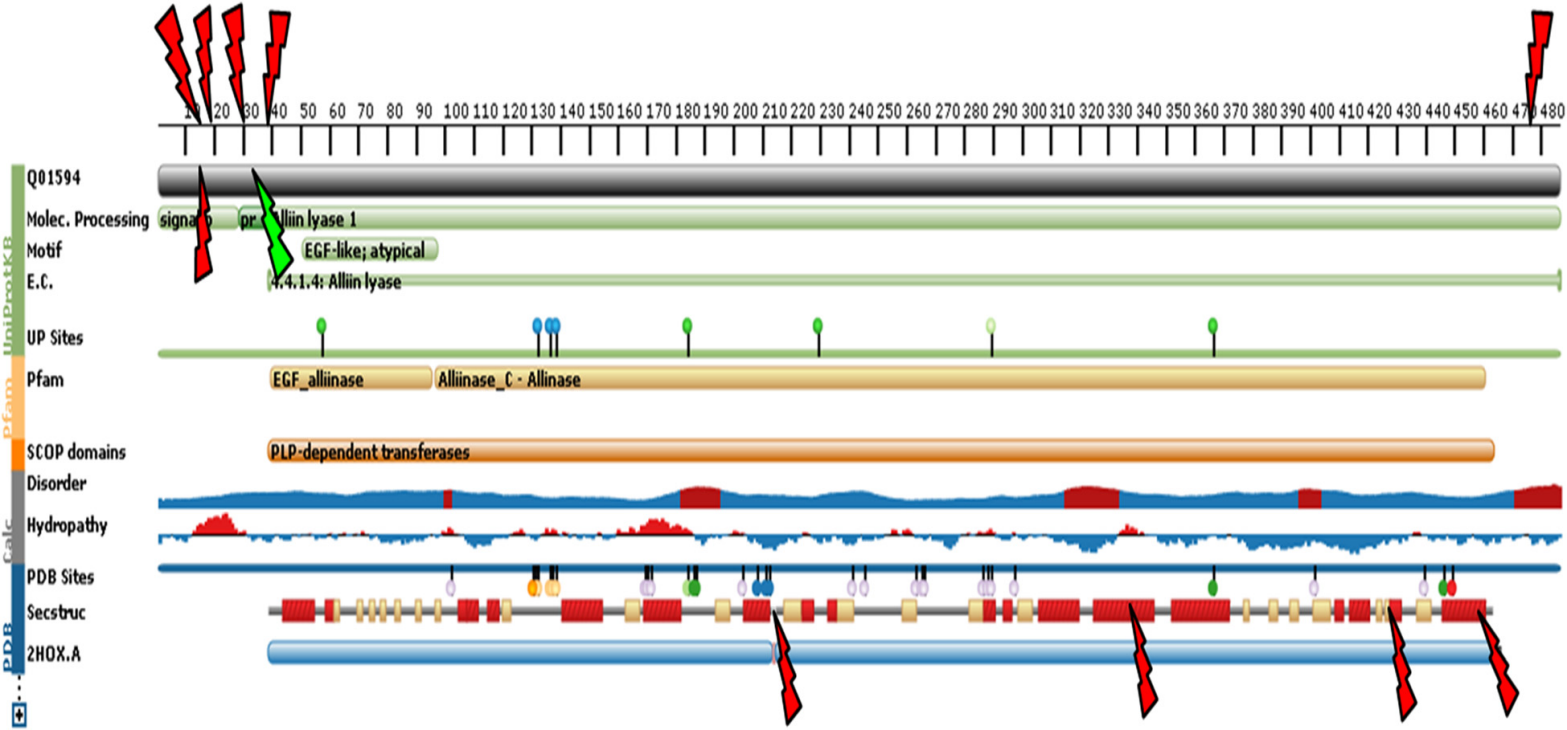
Schematic diagram of the features of the garlic alliinase structure. Schematic diagram indicating the found and mapped amino acid changes [protein feature view of PDB entries mapped to UniProtKB sequence Q01594 (ALLN1_ALLSA)]. red arrows – amino acid changes, green arrow – serine insertion after Asn^33^

Other mutations were found in exons 1, 3, 4 and 5 that altered the protein sequences (Table 2). Silent mutations were also found that did not affect the amino acid sequences due to the degenerate genetic code. The coding sequences of the binding and catalytic domains remained unchanged for all analyzed members of the gene family, indicating that the domains are very conservative (Fig. 1).

**Table 2:** Mutations found in the alliinase aa sequences and their types and positions in the protein (cv. Jovan)

Region	Mutation	Nucleotide position	Type of mutation	AA changes	AA position	SV
**EXON 1**	A↔C	28	Transversion	S↔R	10	SV9
	A↔-	39	In/Del	ORF schift	13	SV9
	C↔G	43	Transversion	P↔A	15	SV2-SV10
	A↔T	58	Transversion	L↔M	20	SV5-SV10
	C↔T	85	Transition	L↔F	29	SV2-SV10
	AGT	100	In/Del	S	33	SV6-SV10
	C↔G	109	Transversion	Q↔E	37	SV5
**EXON 3**	A↔T	1119	Transversion	N↔D	213	SV2-SV10
**EXON 4**	G↔A	1626	Transition	E↔K	381	SV2, SV10
**EXON 5**	A↔G		Transition	N↔S	427	SV2, SV7-SV10
	A↔G		Transition	K↔E	457	SV2-SV4, SV6-SV9
	T↔C		Transition	I↔T	474	SV2-SV10

**Fig. 2:**
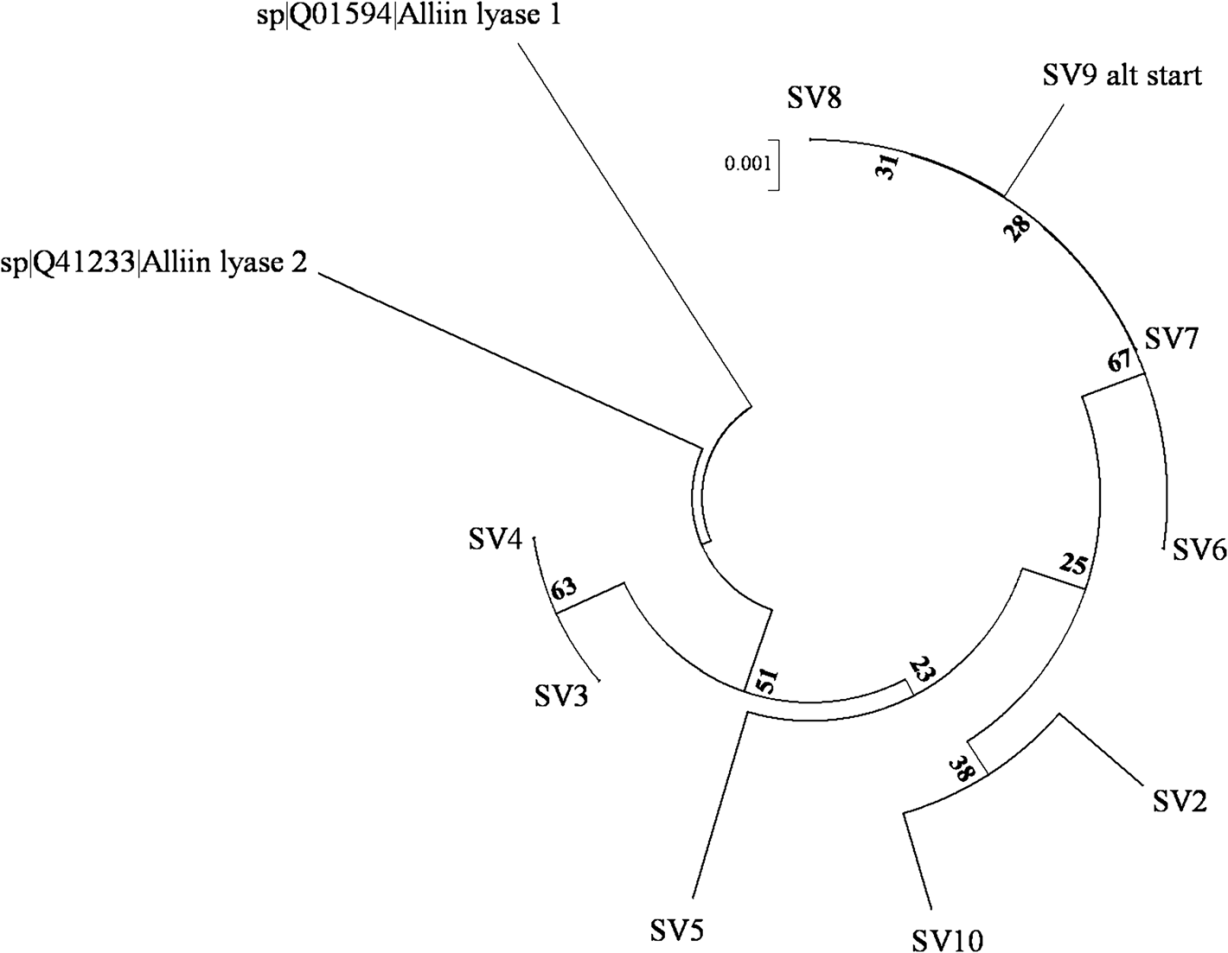
Protein sequence relationships of garlic alliinases from cv. Japo and two complete alliinase sequences. The phylogenetic tree was inferred using the neighbor-joining method [50]. The optimal tree with a branch length sum of 0.03415794 is shown. The percentage of replicate trees in which the associated sequence variants (SVs) clustered together in the bootstrap test (1000 replicates) is shown next to the branches [51]. The tree is drawn to scale, with branch lengths in the same units as those of the evolutionary distances. The evolutionary distances were computed using the Poisson correction method [52] and are represented as the number of amino acid substitutions per site. All ambiguous positions were removed for each sequence pair. There were a total of 487 positions in the final data set. Evolutionary analyses were conducted with MEGA6 [49]

The comparison of nine SVs from cv. Jovan with a reference sequence [GenBank: Z12622, GenBank: gi16108, protein Uni-Prot: Q01594] and alliin lyase 2 protein [Uni-Prot: Q41233] indicated that the obtained sequences were closer to each other than to the reference sequences, which was supported by the bootstrap values indicated in the dendrogram (Fig. 2).

The exon sequences of cv. Japo, Djambul and landrace Marhfeld showed similar features. The catalytic and conservative domains did not contain any mutations. The mutation rates of the vacuolar signal peptide sequences were similar to its rate in cv. Jovan.

### Sequence polymorphisms in introns

The sequences available in the NCBI database and the newly identified intron sequences from two bolting garlics (cv. Jovan and landrace Djambul1), one nonbolting garlic (cv. Japo) and one semibolting garlic (landrace Marhfeld) were used to assess polymorphisms of the intron sequences. A much higher variability compared with the exon sequences was found for the intron regions. We identified insertions/deletions (In/Dels) and simple sequence repeat (SSR) loci which gave rise to intron length polymorphisms (ILPs; Table 3).

**Table 3:** ILPs found in alliinase introns

***1*** ^**st**^ **intro** ***n***	**ILP variant sizes** ***(bp)***										
	195	196	197	199	200	201	202	203	205		Total
Distribution of different sequences	1	1	1	(4 + 1)	(57 + 12 + 6 + 1)	(16 + 9 + 1 + 1)	1	(29 + 3)	1		
Number of Sequences	2	14	14	5	76	27	1	32	8		**179**
**2** ^**nd**^ **intron**	**ILP variant sizes** ***(bp)***										
	**105**	**108**	**109**	**110**	**111**	**112**	**113**	**114**	**115**	**116**	**Total**
Distribution of different sequences	1	1	1	(31 + 16)	(7 + 3 + 1)	1	(23 + 18 + 5)	(4 + 1 + 1 + 1)	1	1	
Number of Sequences	1	1	23	47	11	1	46	7	2	1	
	**ILP variant sizes** ***(bp)***										
	**117**	**118**	**119**	**120**	**121**	**122**	**123**	**124**	**125**	**126**	
Distribution of different sequence	(8 + 1)	1	1	1	(4 + 1)	1	1	1	1	1	
Number of Sequences	9	1	5	1	5	1	5	1	5	1	**174**
***3*** ^**rd**^ **intron**	**ILP variant sizes** ***(bp)***										
	**120**	**124**	**128**	**129**	**132**	**133**					**Total**
Distribution of different sequences	1	(28 + 26 + 7)	(32 + 22 + 8 + 6 + 5 + 4)	1	1	(5 + 19)					
Number of Sequences	5	61	77	10	11	24					**188**
***4*** ^**th**^ **intron**	**ILP variants sizes** ***(bp)***										
	**88**	**89**	**106**	**109**							**Total**
Distribution of different sequences	1	(52 + 30 + 23 + 21)	(30 + 12)	1							
Number of Sequences	1	126	42	2							**171**

147 from NCBI and 24 from cv.Japo, landrace Marhfeld, landrace Djambul1 and cv. Jovan

The first intron ranged from 195 to 205 bp due to the presence of In/Dels and one compound microsatellite with (AC)_4–6_(TA/G)_4_ repeats. The second intron ranged from 105 to 126 bp and contained In/Dels and the compound microsatellite repeats (AT)_4–11_(GT)_3–5_. In the third intron, In/Dels were again detected along with C(TA)_1-3_TT repeats. The ILPs of the third intron ranged from 120 to 133 bp, and up to six sequence variants were found. Only two major variants of ILPs were found in the fourth intron (89 or 106 bp). No variable length SSRs were found in this intron. In addition to length polymorphisms, some nucleotide changes (SNPs) and In/Dels were found even in sequences of the same size (Table 3).

As most ILP variants were identified in the first three introns, we developed a specific PCR assay (for primers, see Table 4) capable of capturing intron length variability. After optimizing the reaction conditions, we performed the assay to assess length polymorphisms in an additional 131 accessions. Thus, the degree of variability in the newly identified ILPs was estimated across numerous garlic accessions.

**Table 4:** Primer sequences used for ILP analysis

Primer	Forward primer (5´- 3´)	Reverse primer (5´- 3´)	Annealing temperature
intr01	**6FAM**-AAGATCCAAGGTTGCTCTGC	CAGCAGCATTTCCCACTACC	63 °C
intr02	**HEX**-ACTCGTCATCTCTCTTTCACC	TGTAATGAGGCCAGTAGTAAACC	65 °C
intr03	**NED**-TTACTACTGGCCTCATTACACC	TTTGAGGAAGCTCTTGATAGG	65 °C

In total, 405 amplicons differing in size were scored across 135 analyzed accessions (see Additional file 1). The data were processed and used for cluster analysis. The cluster analysis divided the accessions into several clusters, reflecting their genetic divergence (see Additional file 2). The first of the clusters consisted of 31 non-bolting *A. sativum* L. genotypes (softneck garlic) originating mostly from central Europe and the former Soviet Union. The second cluster was formed by 42 accessions (both nonbolting and semibolting) from southern Europe. These two clusters were the most numerous and encompassed over 60 % of all analyzed accessions. Members of each clusters contained almost identical ILPs. This finding reflected the low level of intron variability among these garlic types. The bolting accessions constituted several groups, and the clustering was coupled with the individual places of origin of the accessions. For example, one cluster originated in the Czech Republic, Slovakia and Poland, and the other two clusters contained accessions from central Asia. These results indicate that the alliinase ILPs accurately reflect the bolting types of the analyzed accessions and could be used for the identification of garlic genotypes together markers, such as SSRs.

## Discussion

Alliinase, which is encoded by members of a multigene family, is an important enzyme involved in garlic defense against pests [15, 16]. It is also associated with the organoleptic properties of garlic [17, 18]. The variability of its encoding loci have been described to some extent, but more information is needed. Namely, the number of alliinase loci within the garlic genome is still uncertain. *Allium* species, including garlic, are diploid and have extremely large genomes that contain numerous multicopy genes, duplications and noncoding sequences [19, 20]. It has been shown that even gene duplication allows for the better adaptation of plant species to the surrounding environment (for a review, see [21]). Because garlic solely undergoes vegetative propagation [22], gene multiplication may play an important role in its adaptability to different environments. This phenomenon has been already described in general terms elsewhere [23]. Hence it is necessary to elucidate the variability of the alliinase gene family.

We identified the DNA sequence variants of alliinase in cv. Jovan, which we investigated in more detail. Because previous studies have not focused on the first exon of the alliinase gene [24, 25], we compared our sequences to complete sequences available in databases. Only the first ATG of sequence [GenBank: Z12622], which was used as a reference, was located in the same position as in our sequences. The start codons of the other two available NCBI sequences [GenBank: S73324 and FJ786257.1], were located at different positions, which altered the sizes and functions of the resulting signal peptides. Only one sequence characterized in our experiment possessed an alternative start codon or was a nonsense sequence. For the remaining 104 partial sequences available in NCBI with unidentified start codons, almost the entire 5’ regions of exon I encoding the signal peptides (SPs) were missing.

We found that exon I was more polymorphic compared to the exons coding for structural proteins. Mutational changes in the signal peptide region seemed to be rather well tolerated. Irrespective of the sequence changes, the polymorphisms of the predicted signal peptides in our sequences were found to maintain the required signatures for the targeting of the premature protein to the secretory pathway [26–28]. We showed that exon I of the other three analyzed accessions (Djambul1, Marhfeld and Japo) retained the same sequence variants as cv. Jovan. We thus consider these sequence variants to be confirmed.

No alterations were found in the catalytic and/or binding domains of the mature proteins [10]. No sequence alternations were found to affect any cysteines, which allowed for the maintenance of the tertiary protein structures by disulfide bonds [8]. This finding could be expected for a protein with high substrate specificity participating in a catalytic process. Endo et al. [24] reported that only functional copies of this gene occur in analysed genotypes garlics, probably reflecting the use of commercial garlic samples with fully functional enzymes to ensure the metabolism of flavor precursors. They also found the exon sequences highly conserved among all the clones studied. However, they did not reported sequences of more than two gene copies per a genotype and did not include complete 5´ends. Thus they did not focused on appropriate protein targeting which correlate with expected function of the protein. As lack of the protein activity have not been reported for commercially used garlic cultivars this feature need not to be investigated in more details.

We also investigated polymorphisms in the introns of this gene family. We demonstrated a greater number of polymorphisms in the intron regions, with 39 variable sites in four introns compared with only 12 variable sites in the exons of one accession (cv. Jovan). This finding is similar to the expected ratio and is in line with reports involving other *Allium* genes [29]. Similar variability ratios were found for the other three accessions used in our alliinase variability analysis (Djambul1, Marhfeld, and Japo). In addition, this finding is in accordance with the sequences retrieved from the NCBI database.

Based on the polymorphic data, we attempted to estimate the number of gene family members in cv. Jovan. We identified nine DNA sequence variants in Jovan (primers ALL_total_) and an additional five different partial sequences (primers ALL_part_). The assessment of variability in the alliinase gene family indicated the presence of up to 14 sequence variants in diploid genome of cv. Jovan. Cavagnaro et al. [30] have detected at least 4–8 gene copies depending on the garlic accession by Southern blot, whereas the Polymerase Chain Reaction (PCR) amplification and sequencing of an intron-bearing fragment revealed up to 27 sequence variants in the same accessions. The authors concluded that tandem duplications in distinguishable by Southern hybridization could lead to an underestimation of the copy number. On the other hand, PCR and sequencing may have led to the overestimation of the gene copy numbers. We suggest that other shortcomings of these techniques also contributed to their findings [31]. Our data indicated that the number of sequence variants lies somewhere in between both of the a forementioned values. However, the accessions may differ in this respect. Moreover, the authors of sequences deposited in NCBI databases did not consider the polygenic nature of this family thus, the available information cannot be used for copy number estimation. Digital PCR could be therefore recommended as a precise tool that can be used to answer this question [32].

Several molecular marker systems have been proposed for germplasm analysis [33, 34], (for a review, see [35]), who exploited the a forementioned high intron variability to develop a new assay for analysis of garlic genetic resource diversity). However, our assay allows for the screening of garlic cultivars for ILPs and was validated using 132 accessions, indicating its robustness and reproducibility. The high degree of polymorphisms identified in the different accessions suggests their applicability for genetic resource variability screening.

Cluster analysis based on the ILP markers demonstrated the genetic similarities among the analyzed accessions and well separated them according to their bolting types (Fig. 2). Similarily Ipek et al. [36] developed a bolting marker based on variability found in a chimeric region of garlic mitochondrial genome. The ILPs presented in this study divided garlic clones not only according their bolting types, but surprisingly ILPs subcluster them in a similar way as previously published AFLP analysis [14], in which nearly the same accessions were evaluated. The nonbolting accessions from AFLP clusters 3, 4 and 5 were rich in alliin. These same accessions associated together according to the alliinase ILPs. Similarly, a mixture of non-bolting and semi-bolting garlic accessions belonging to the AFLP clusters 6, 7 and 8, which were characterized by low alliin content, had the same ILP patterns. These accessions have probably not locally adapted since coming from Romania and the Mediterranean region of Europe. The bolting garlic accessions, which formed several specific AFLP sub-clusters, each of which had a typical alliin content, formed similar clusters according to the ILP data as were observed by AFLP analysis. The variability within the alliinase gene family found for bolting garlic was higher compared with that identified for the non-bolters or semi-bolters. The bolting garlic accessions were subdivided into several subclusters with fewer members than the nonbolting and semibolting groups as a result of their higher diversity. We assume that bolting garlic might have propagated via sexual reproduction for a longer time, thus acquiring a higher degree of diversity due to recombination. Because even bulbils on stolons are involved in the multiplication/reproduction of garlic, it is also possible that this greater diversity is due to the higher mutation rates of the propagating material.

In this study, we have proven that the polymorphisms identified with ILP markers are associated with garlic bolting types and consequently with alliin and methiin contents or their ratio. This newly developed assay allows for the recognition of phenotypes with desirable properties and can be easily used by breeders to benefit breeding programs.

## Conclusions

We confirmed multigenic nature of alliinase family and found 9 copies of the gene within Czech garlic cultivars Jovan. We estimated that the minimum number of gene copies in the diploid genome was fourteen. Exon sequences coding for functional part of the protein were highly conserved. Polymorphism was found in sequence coding pro signal peptide. High polymorphism was found in introns. That allow us to develop ILPs markers. The markers separated bolting and nonbotling genotypes. The obtained profile generated for individual genotypes clustered them in a similar way as previously AFLP analysis did. It reflected also alliin content. Thus the newly developed ILPs marker could be used in breeding programmes.

## Methods

### Plant materials and genomic DNA extraction

Four garlic genotypes, cv. Japo, landrace Marhfeld, landrace Djambul1 and cv. Jovan, representing basic morphological types, were used to obtain full genomic sequences of alliinase and to detect possible polymorphic sites. Altogether, 135 genotypes from the genebank of the Crop Research Institute, Prague, Czech Republic were further analyzed to assess polymorphisms across a larger set of clones (see Additional file 1). Genomic DNAs were isolated from leaves using the CTAB protocol [37]. DNA quantities and qualities were verified spectrophotometrically at 260/280 nm and by gel electrophoresis.

### Amplification and cloning of alliinase DNA coding sequences

The amplification of the alliinase genomic sequences of cv. Jovan was performed with a set of primers (ALL_total_ and ALL_part_) designed with the web-based Primer3 software http://frodo.wi.mit.edu/ [38] using known DNA sequences (*A.sativum* L. mRNA encoding precursor alliinase sequence, [GenBank:Z12622] and *A.sativum* L*.* alliinase mRNA partial coding sequences [GenBank:AF409952]. Three primer pairs (ALL_part1_, ALL_part2_, ALL_part3_) were used to obtain partially overlapping DNA sequences.

The amplification was performed with a DNA Thermal Cycler Flexigene, Techne FFG02HSD (AFAB Lab Resources, Frederick, USA) under the following conditions: the initial activation of *Taq* polymerase at 95 °C for 5 min, followed by 35 cycles of 95 °C for 30s, 65 °C for 40s, 72 °C for 1 min and 72 °C for 15 min for primer pair ALL_parts_. For primer pair ALL_total_, cycling conditions were 95 °C for 5 min followed by 35 cycles of 95 °C for 60s, 63 °C for 1 min 30s, 72 °C for 2 min 30s and 72 °C for 15 min. Each 50-μL reaction contained 200 μM dNTP, 1.5 mM MgCl_2_, 1 × PCR Buffer, 2.5 U Taq DNA polymerase (Qiagen, Hilden, Germany), primers at concentrations 0.2 μM and 100–200 ng of genomic DNA.

The polymerase chain reaction (PCR) products were separated by gel electrophoresis and cloned into a plasmid PCR 2.1 TOPO TA-cloning vector (Invitrogen, Foster City, USA). The plasmids were transformed into *E. coli,* and colonies carrying recombinant plasmids were identified according to the manufacturer’s instructions. DNA was isolated using a High Pure Plasmid Isolation Kit (Roche, Basel, Switzerland).

### Sequencing reactions and sequence analyses

Sequencing reactions were performed using a BigDye Terminator Cycle Sequencing Kit v.3.1, according to the Applied Biosystems protocol (Foster City, USA). The data were analyzed using Applied Biosystems DNA Sequencing Analysis Software V5. 2.

The sequences were subsequently analyzed using BLAST tools [39] and compared with CLUSTAL-W software http://www.ebi.ac.uk/Tools/msa/clustalw2/ [40] and MEGA version 5.2 http://www.megasoftware.net/ [41].

Plant alliinase nucleotide sequences with high similarity (E-value <0.02) to those obtained from cv. Jovan were retrieved from GenBank http://www.ncbi.nlm.nih.gov/ based on the BLASTn results [39]. The exons and introns of the genomic sequences were identified by comparing our sequences with *A.sativum* mRNA encoding the precursor alliinase sequence [GenBank: Z12622.1] using SPLIGN software http://www.ncbi.nlm.nih.gov/sutils/splign/splign.cgi [42] and MEGA software version 5.2 http://www.megasoftware.net/ [41].

### Characterization of derived protein sequences and post-translational modification prediction

The open reading frame (ORF) sequences of the putative alliinase genes obtained in our analyses and other sequences (based on the BLASTn results) were translated into amino acid sequences using ExPASy (Expert Protein Analysis System) Translate Tool [43], which is a proteomics server of Bioinformatics and/or MEGA5.2 software. The deduced amino acid sequences were subjected to motif analyses, and the presence of alliinase domains was verified using InterPro Scanonline, an integrated search in PROSITE [44], Pfam [45], PRINTS and other family and domain databases.

Putative N-terminal signal peptides were identified using SignalP server http://www.cbs.dtu.dk/services/SignalP/ TargetP 1.1 server was used to predict the subcellular localizations of the eukaryotic proteins [46] and the locations of the cleavage sites [47, 48].

### Intron length polymorphisms and analysis of their variability

Primers spanning the most polymorphic introns were designed by Primer 3.0 software http://frodo.wi.mit.edu/ [38] using the conserved sequences from two neighboring exons. Amplification was performed in a volume of 15 μl containing genomic DNA, 1.0 unit of *Taq* DNA polymerase and 2 mM MgCl_2_ in a 1x PCR reaction buffer (Qiagen, Hilden, Germany), 100 μM dNTPs (Invitrogen, Foster City, USA), 0.2 μM each of the forward and reverse primers and 100 ng of genomic DNA. PCR was carried out with a Techne FlexigeneDNA thermal cycler (AFAB Lab Resources, Frederick, USA) that was programmed as follows: 5 min at 94 °C, followed by 35 cycles of 1 min at 94 °C, 40 s at 65 °C and 40 s at 63 °C (depending on the primers) and 72 °C for 5 min. First, unlabeled primers were used for the amplification of the expected polymorphic regions for the four genotypes (cv. Japo, landrace Marhfeld, landrace Djambul1 and cv. Jovan). The products were cloned as described above and sequenced again to verify the results. Then, labeled primer pairs were used to investigate polymorphisms in all 135 genotypes.

The 5‘end of the forward primer of INT_F1 was labeled by 6-FAM, INT_F2 with HEX and INT_F3 with NED (Applied Biosystems, Foster City, USA). A total of 0.4 μl of PCR product that was diluted ten-fold with water was mixed with 10 μl of Hi-Di formamide containing 1 μl of GeneScan 500 LIZ- internal size standard (Applied Biosystems, Foster City, USA). The heat-denatured products were run on an ABI Prism 3130 Genetic Analyzer, and the allele data were analyzed with Gene Mapper (Applied Biosystems, Foster City, USA).

### Polymorphic region data analysis

The data from Gene Mapper were compiled into spread sheets. For each locus, the presence or absence of bands in each size category throughout all genotypes was scored. The data were set in a binary matrix. Phylogenetic and molecular analyses were conducted using MEGA version 5.2 http://www.megasoftware.net [41].

Genetic similarities were calculated using Jaccard coefficients. A dendrogram was constructed by clustering according to the unweighted neighbor-joining (UNJ) method using MEGA6 http://www.megasoftware.net/ [49].

### Single nucleotide polymorphisms, insertions and deletions

Whole genomic sequences and partial genomic sequences of alliinase of cv. Jovan and sequences available in the NCBI database were compared to detect putative single nucleotide polymorphism sites and insertions/deletions using BLASTn [39], CLUSTALW http://www.ebi.ac.uk/Tools/msa/clustalw2/ [40] and MEGA software http://www.megasoftware.net [41].

### Availability of supporting data

The data sets supporting the results of this article are included within the article and its additional files. The phylogenetic tree supporting the results of this article is available in the TreeBase repository, http://purl.org/phylo/treebase/phylows/study/TB2:S17557. Sequence are available in GenBank (accession no. KR270349 - KR270357).

## Additional files

Additional file 1: Table S1. List of analyzed varieties of garlic (*Allium sativum* L.). ^1^Evigez = Czech Plant Genetic Resources Documentation System. ^2^ Type: SB = semi-bolting, NB = non-bolting, and B = bolting. ^3^Ori = origin of donator: AUT = Austria, BGR = Bulgaria, CZE = Czech Republic, FRA = France, ITA = Italy, KOR = Korea, POL = Poland, ROM = Romania, PRT = Portugal, SUN = former Soviet Union, and SVK = Slovakia.

Additional file 2: Figure S1. Dendrogram for the 135 (*Allium sativum* L.) genotypes based on ILP markers. Dendrogram was constructed by DARwin 5.0 using the simple matching (SM) dissimilarity index and unweightedneighbor-joining (UNJ) method The robustness of the nodes of the dendrogram was tested by bootstrap analysis using 1,000 resamplings. The resulting dendrogram was drawn by iTOL Version 2.1 HYPERLINK http://itol.embl.de/index.shtml [53]. Note: black=nonbolting type, lilac=semibolting type, and yellow=bolting type.

## Abbreviations

aa: Amino acid
AFLP: Amplified fragment length polymorphism
Asn: Asparagine
cDNA: Complementary DNA
Cv: Cultivar
Cys: Cysteine
dNTP: Deoxynucleotide-5/-triphosphate
EST: Expressed sequence tag
GSS: Genomic survey sequence
ILP: Intron length polymorphism
In/Dels: Insertion/deletions
MgCl2: Magnesium chloride
mM: Milimolar
μL: Microliter
μM: Micromolar
ng: Nanogram
nt: Nucleotide
ORF: Open reading frame
PCR: Polymerase chain reaction
pH: Potential of hydrogen
R-SOH: Sulfenic acid
SSR: Simple sequence repeat
SV: Sequence variant
UNJ: Unweighted neighbor-joining
UTR: Untranslated region
