# Supplementary figures and images for: Garlic (A. sativum L.) alliinase gene family polymorphism reflects bolting types and cysteine sulphoxides content
Source: BMC Genet. 2015 May 22;16:53. doi: 10.1186/s12863-015-0214-z (PMC4440563; doi:10.1186/s12863-015-0214-z)

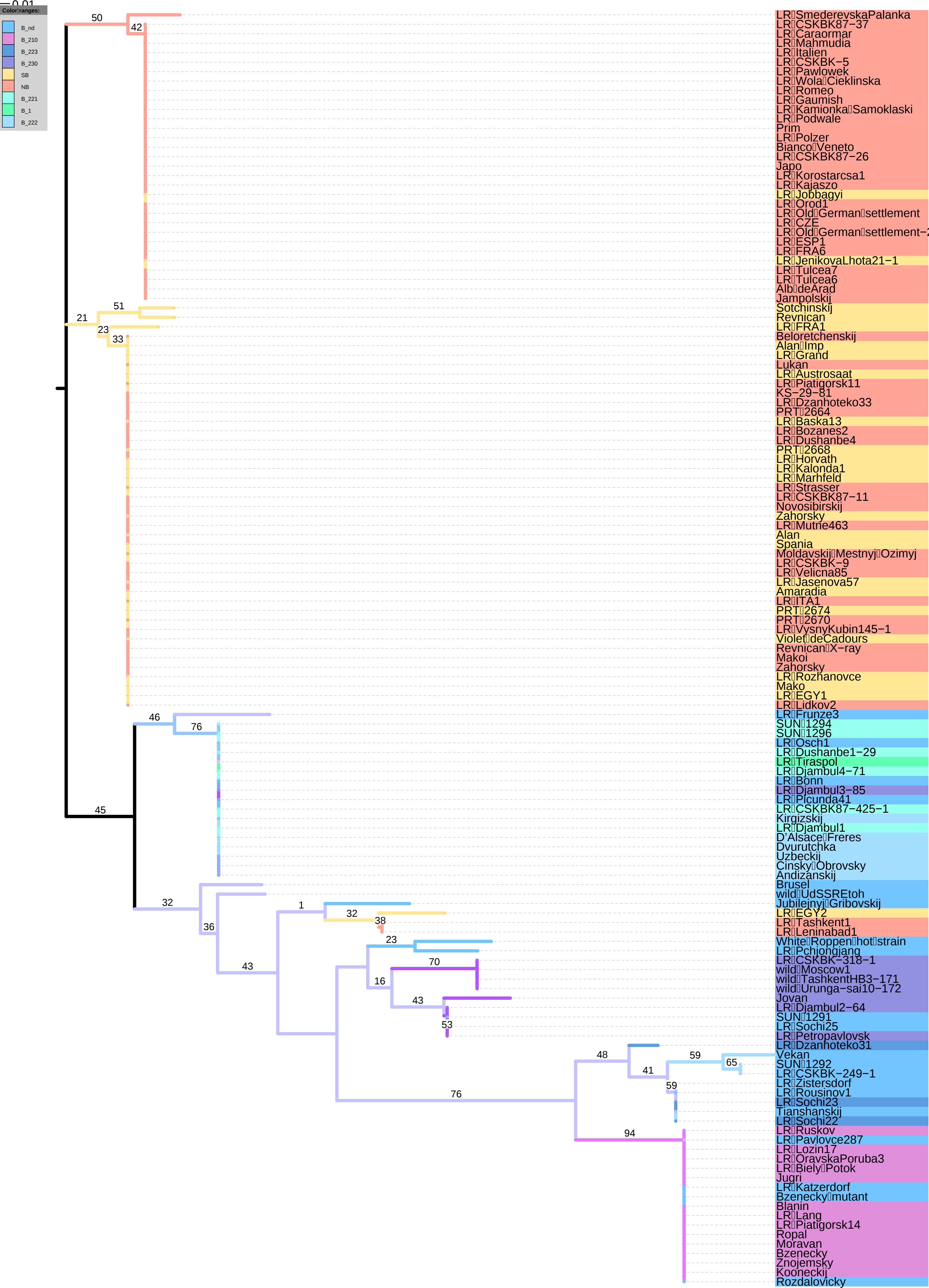

Supplement: Supplementary file 2 — Dendrogram for the 135 (Allium sativum L.) genotypes based on ILP markers. Dendrogram was constructed by DARwin 5.0 using the simple matching (SM) dissimilarity index and unweightedneighbor-joining (UNJ) method The robustness of the nodes of the dendrogram was tested by bootstrap analysis using 1,000 resamplings. The resulting dendrogram was drawn by iTOL Version 2.1 HYPERLINK http://itol.embl.de/index.shtml [53]. Note: black=nonbolting type, lilac=semibolting type, and yellow=bolting type. [file 12863_2015_214_MOESM2_ESM.pdf]
